# The Finger Feeding Method and Relactation

**DOI:** 10.7759/cureus.24044

**Published:** 2022-04-11

**Authors:** Nalan Karabayir, Edanur Mertturk Potak, Sümeyye Karaman, Muhammed Fatih Sebirli, Mustafa Beykan Istanbullu, Mehmet Potak, Burcu Gizem Teber

**Affiliations:** 1 Pediatrics Department, Istanbul Medipol University, Istanbul, TUR

**Keywords:** induced lactation, relactation, bottle feeding, supplementary nursing system, finger-feeding, breastfeeding

## Abstract

Background: Breastfeeding is one of the most important factors ensuring the healthy growth and development of babies. Preterm babies, babies with metabolic, neurological, or developmental delays, babies separated from their mothers for any reason, and adopted babies need alternative feeding methods. This study was carried out to investigate the effect of the finger feeding (FF) method on relactation.

Method: In this retrospective study, infants who were admitted to the Istanbul Medipol University breastfeeding counseling clinic between January 2020 and June 2021 and were recommended to be fed with finger feeding were evaluated. Gender, gestation, mode of delivery, birth weight, age, cause for admission, feeding type, breastfeeding starting time, finger feeding time, and breastfeeding duration of the cases were recorded from the counseling forms.

Results: Of 41 babies, 29.3% were girls and 70.7% were boys, and 82.9% were term. Seventeen (41.5%) were born with spontaneous vaginal delivery and 24 (58.5%) with a cesarean section. The most common reason for admission was found to be the inability to latch. While 30 (73.2%) of the babies fed with finger feeding were able to suck, nine babies continued to be fed with a bottle, one baby with a spoon, and one baby with a supplemental nursing system (SNS). The breastfeeding starting time was 23.1 ± 27.5 (1-100) days.

Conclusion: The finger feeding method is an effective alternative feeding method for successful breastfeeding. There is a need for studies to be conducted with more babies, both preterm and term, in this regard.

## Introduction

Human milk is the most suitable nutrient for term and preterm babies. Although it is known that the ideal way for babies to get breast milk is to suckle from the breast, preterm infants, babies with a cleft palate or other anomalies, babies with metabolic, neurological, or developmental delays, and babies separated from their mothers for any reason, including adopted babies, may have difficulties with breastfeeding [1]. Also, alternative feeding methods are of great importance in relactation and induced lactation. In these cases, a need for alternative feeding methods arises, and bottles and nasogastric tubes are often used to feed babies in neonatal and maternity units [2].

Alternative feeding methods are cup feeding, spoon feeding, syringe feeding, supplemental nursing system (SNS), finger feeding (FF), and baby-led bottle feeding. The ideal method should be suitable for the baby's physical characteristics and maturity level, contain equipment that parents can easily apply, provide, and clean, and help the baby learn to breastfeed. Alternative feeding methods are contraindicated if the baby does not have a sucking, swallowing, or gag reflex [3]. One of the alternative feeding methods, finger feeding, is used when the baby fails to suckle or when breastfeeding needs to be stopped temporarily. This study was carried out to investigate the effect of the FF method on relactation.

## Materials and methods

In this study, babies who were recommended to be fed with FF in the breastfeeding counseling clinic of Istanbul Medipol University Social Pediatrics clinic between 2019 and 2021 were evaluated retrospectively. In our center, the FF method is recommended as the first option in relactation, with other alternative feeding methods being offered to mothers who do not accept or cannot do this method.

Gender, gestation, mode of delivery, birth weight, age, cause for admission, feeding type, lactation gap (duration of lactation interruption), bottle or SNS use, breastfeeding starting time, duration of finger feeding, and breastfeeding success were recorded from the counseling forms. The feeding styles of babies who could not be breastfed alone and the total breastfeeding time of babies who were successfully breastfed were obtained by telephone interview with the mother.

In the descriptive statistics of the data, mean, standard deviation, median minimum, maximum, frequency, and ratio values were used. The distribution of variables was measured with the Kolmogorov-Smirnov test. The Mann-Whitney U test was used in the analysis of quantitative independent data. The Chi-square test was used in the analysis of qualitative independent data, and Fisher’s exact test was used when the Chi-square test conditions were not met. SPSS 27.0 program was used in the analysis.

The approval of the ethics committee of the study was obtained from the ethics committee of Istanbul Medipol University.

## Results

A total of 41 term babies, 12 girls and 29 boys, were included in our study. Seventeen (41.4%) were born with normal spontaneous vaginal delivery, and 24 (58.6%) were born with cesarean section delivery. The mean birth weight of the babies was 3073.1 ± 731.9 g (2320-4430), and the mean age was 51.3±38.5 (3-173) days (Table1).

**Table 1:** Features of the babies *Finger feeding method **Supplemental nursing system

	Min-max	Median	Mean ±SD or n (%)
Age (d)	3–173	41.5	51.3±38.5
Gender			
Girl			12 (29.3)
Boy			29 (70.7)
Gestation week	28–40	38	37.5±2.8
Mode of delivery			
Spontaneous vaginal			17 (41.5)
Cesarean section			24 (58.5)
Birth weight (g)	1170–4530	3180	3073.1±731.9
Feeding type on admission			
Formula			1 (2.4)
Breast milk			10 (24.4)
Breast milk + formula			30 (73.1)
Lactation gap (d)	3–94	18	61.45±2.8
Total FF* usage time (d)	1–180	15	38.7±44.1
Breastfeeding with FF*			
No			11 (26.8)
Yes			30 (73.2)
Breastfeeding starting time (d)	1–100	8.5	23.1±27.5
Bottle use			
No			29 (70.7)
Yes			12 (29.3)
SNS**			
No			39 (95.1)
Yes			2 (4.9)
Feeding type after FF* usage			
Breastfeeding			30 (73.2)
Bottle use			9 (22)
Spoon feeding			1 (2.4)
SNS**			1 (2.4)

The main reasons for admission were shown in Table 2.

**Table 2:** Causes of breastfeeding problem

	n (%)
Inability of latching	25 (60.9)
Refusal to breastfeeding	6 (14.6)
Mastitis	3 (7.3)
Nipple crackles	3 (7.3)
Tongue tie	3 (7.3)
Engorgement	1 (2.4)
Mammary reduction operation	1 (2.4)
Adopted baby	1 (2.4)

Ten babies were fed with expressed breast milk, 30 babies with expressed breast milk and formula milk, and one baby with only formula milk. While lactation started in 30 (73.2%) of finger-fed infants, 9 of 11 cases (one of whom was a preterm baby) continued to be fed with breast milk with a bottle, one baby with a spoon, and one baby with SNS. The mean breastfeeding starting time was 23.1 ± 27.5 (1-100) days, while the duration of finger feeding was 75.57 ± 44.59 days (1-180) (Table 3).

**Table 3:** Comparison of groups *Finger feeding method **Supplemental nursing system ^a^Mann-Whitney U test ^b^Chi-square test

	Successful relactation (−)		Successful relactation (+)		p-Value
	Mean ± SD or n (%)	Median	Mean ± SD or n (%)	Median	
Age (d)	48.9±28.8	45	52.2±42	41	0.844^a^
Gender					
Girl	3 (27.3)		9 (30)		0.865^b^
Boy	8 (72.7)		21 (70)		
Gestation week	37.9±1.5	38	37.3±3.1	38	0.988^a^
Mode of delivery					
Spontaneous vaginal	5 (45.5)		12 (40)		0.753^b^
Cesarean section	6 (54.5)		18 (60)		
Lactation gap (day)	36.1±33.2	18	29.6±22.4	30	0.789^a^
Birth weight (g)	3325.5±615.8	3140	2977.4±759.1	3220	0.431^a^
Feeding type on admission					
Formula	1 (9.1)		0 (0)		0.268^b^
Breast milk	2 (18.2)		8 (26.7)		0.700^b^
Breastmilk + formula	8 (72.7)		22 (73.3)		0.719^b^
Total FF* usage time	59.6±49,8	60	31.0±40.1	14	0.018^a^
Bottle use					
No	6 (54.5)		23 (76.7)		0.117^b^
Yes	5 (45.5)		6 (23.3)		
SNS**					
No	10 (90.9)		29 (96.7)		0.470^b^
Yes	1 (9.1)		1 (3.3)		

## Discussion

In our study, 73.2% of babies were successful in breastfeeding with the finger feeding method. The most common reason for admission was the inability to latch.

Babies may have sucking problems for a variety of reasons. Especially, preterm babies are not always successful in breastfeeding in their first experience at the breast [4,5]. It is necessary to use alternative feeding methods until breastfeeding begins. The finger feeding method is accepted as a physiological method that facilitates the transition to breastfeeding and improves sucking and respiratory coordination. In addition, finger feeding, which facilitates the correct use of oral muscles, is applied to infants who cannot be applied SNS, who cannot grasp the breast, or when breastfeeding is temporarily contraindicated [3]. In our study, the most common cause was a latching problem.

In a study from Turkey, it was determined that the finger feeding method is an effective method for sucking skills, transition to breastfeeding, and hospital stay in preterm infants [6]. In a study of preterm infants, finger feeding was shown to be a better transitional feeding method in terms of efficacy compared to cup feeding due to lower milk loss and fewer complications [7]. Oddy et al. detected that infants fed by finger-feeding had fewer physiological stress symptoms, better comfort levels, and earlier development of sucking and swallowing functions [8]. In our study with term babies, lactation started in 73.2% of infants.

With the finger feeding method, most babies start to suckle from the breast within a week, but in some cases, it may take several weeks [9,10]. In our study, the median duration of starting suckling was 23.1 ± 25.7 (1-100) days.

Studies have identified success factors for relactation, including maternal motivation, nipple stimulation, family (especially the partner’s), and health professionals' support [11]. Relactation success is also related to the baby's age and nonlactation interval. If the baby is less than two months old, the success rate increases, while if the baby is older than four months, the success rate decreases to 60% [12]. In our study, the average age of the babies was found to be 51.3 ± 38.5 days. It is known that the non-lactation interval being shorter than 30 days is a factor that increases the success of relactation [13]. In our study, no difference was found between the two groups in terms of the lactation gap. 

To the best of our knowledge, our study is the first to investigate finger feeding in term babies. The limitation of our study is that it is retrospective.

## Conclusions

Finger feeding is an effective alternative method that facilitates feeding of the baby from the breast. FF can be recommended for mother-baby couples in relactation and babies who have had latching problems. The main factors affecting the success of FF are the motivation of the mother, the short lactation gap and the baby's age less than three months. In this difficult process, the support of health professionals, family and close friends, especially the father, is very important. More randomized, controlled, large-scale studies with both term and preterm infants are needed to investigate the efficacy of FF. 
